# Genotype imputation accuracy with different reference panels in admixed populations

**DOI:** 10.1186/1753-6561-8-S1-S64

**Published:** 2014-06-17

**Authors:** Guan-Hua Huang, Yi-Chi Tseng

**Affiliations:** 1 Institute of Statistics, National Chiao Tung University, 1001 University Road, Hsinchu 30010, Taiwan

## Abstract

Genome-wide association studies have successfully identified common variants that are associated with complex diseases. However, the majority of genetic variants contributing to disease susceptibility are yet to be discovered. It is now widely believed that multiple rare variants are likely to be associated with complex diseases. Using custom-made chips or next-generation sequencing to uncover the effects of rare variants on the disease can be very expensive in current technology. Consequently, many researchers use the genotype imputation approach to predict the genotypes at these rare variants that are not directly genotyped in the study sample. One important question in genotype imputation is how to choose a reference panel that will produce high imputation accuracy in a population of interest. Using whole genome sequence data from the Genetic Analysis Workshop 18 data set, this report compares genotype imputation accuracy among reference panels representing different degrees of genetic similarity to a study sample of admixed Mexican Americans. Results show that a reference panel that closely matches the ancestry of the study population can increase imputation accuracy, but it can also result in more missing genotype calls. Having a larger-size reference panel can reduce imputation error and missing genotype, but the improvement may be limited. We also find that, for the admixed study sample, the simple selection of a single best-reference panel among HapMap African, European, or Asian population is not appropriate. The composite reference panel combining all available reference data should be used.

## Background

Large-scale genome-wide association studies (GWAS) based on common variants (a minor allele frequency [MAF]≥5%) genotyping have only identified a small fraction of the heritable variation of complex diseases. One explanation may be that many rare variants (MAF <5%), which are not included in the common genotyping platforms (e.g., Affymetrix GeneChip array, Illumina Infinium Beadchip), may contribute substantially to the genetic variation of these diseases [1,2]. Using custom-made chips or next-generation sequencing to uncover the effects of rare variants on the disease can be very expensive with current technology. Consequently, many researchers use the genotype imputation approach to predict the genotypes at these rare variants that are not directly genotyped in the study sample [3]. These predicted genotypes can then be used to test for association, increasing the power and the ability to resolve the causal variants.

Imputation methods work by combining a reference panel of individuals genotyped at a dense set of single-nucleotide polymorphisms (SNPs) with a study sample genotyped at a subset of these sites [4]. These imputation methods first phase the genotypes in the study sample and then look for perfect or near-perfect matches between the resulting haplotypes and the corresponding partial haplotypes in the reference panel; matched haplotype patterns in the reference panel are used to predict unobserved genotypes in the study sample. In this context, one important question is how to choose a reference panel that will produce high imputation accuracy in a population of interest [5,6]. Most imputation analyses have used reference panels composed of haplotypes from public databases, like HapMap 3 and the 1000 Genomes Project. These human genetic variation resources include individuals from a variety of sampling locations in Africa, Asia, and Europe. One might only include the individuals who most closely match the ancestry of the study population as the reference panel [7]. This "best match" strategy reduces the computational burden of imputation, but it can yield suboptimal accuracy with using partial information of diverse reference collections, or in studies with no clear reference matches (e.g., admixed populations) [6]. For this latter situation, Huang et al [6] suggested generating a "weighted mixture" of the available reference data. In contrast to the match approach, Howie et al [5] demonstrated that larger and more diverse reference collections could actually make it easier to identify haplotype sharing with simple models, thereby making imputation faster and more accurate. Some researchers have adopted a 2-stage approach for genotype imputation, in which a subset of individuals is selected for next-generation sequencing, and the obtained whole genome sequence (WGS) data is used as the reference panel, together with the study sample made up of the remaining samples genotyped on commercial genome-wide SNP arrays [8]. This 2-stage approach creates a reference panel that is genetically similar to the study sample and can greatly increase the imputation accuracy, but comes at the extra cost of next-generation sequencing. Several studies [6,8] have compared and discussed various choices of reference panels.

In this report, we analyze 464 individuals with both WGS data and Illumina Infinium Beadchip GWAS data from the Genetic Analysis Workshop 18 (GAW18) data set. The objective is to compare genotype imputation accuracy when adopting different reference panels. Because the participants in the GAW18 data set are Mexican Americans who are an admixed population with differing degrees of Native American, European, and, potentially, African ancestry in each individual, this creates difficulty in selecting the ancestry-matched reference panel. Our results can thus provide evaluation of imputation accuracy in studies of admixed populations.

## Methods

### Data

The GAW18 data set was drawn from a complex pedigree-based study designed to identify rare variants influencing susceptibility to type 2 diabetes on 1043 individuals from 20 Mexican American pedigrees. The GAW18 data set included WGS data for 464 individuals who meet SNP quality control criteria. Approximately 24 million SNPs passing a SNP filtering pipeline (see GAW18 data description files) were identified in these 464 individuals. GWAS data obtained using different versions of the Illumina Infinium Beadchips were provided for 959 individuals, including 464 with WGS, for 472,049 SNPs on odd-numbered autosomes.

This report focuses on analyzing 464 individuals with both WGS and GWAS data. To maximize the available sample size, genetically related individuals were not excluded from our analysis. We only imputed SNPs on chromosome 3. For WGS SNP data, we first obtained the IDs of WGS SNPs that passed support vector machine (SVM) and insertion/deletion (INDEL) proximity filters and were cleaned of Mendelian errors from the file chr3-geno.csv.gz. We then extracted their corresponding genotypes from the file chr3-seq.vcf.gz. We included GWAS SNP genotypes that passed standard quality control procedures and were cleaned of Mendelian errors from the file chr3-gwas.csv.gz.

### Accuracy comparison among different reference panels

The objective is to compare genotype imputation accuracy when adopting different reference panels. Among 464 individuals with both WGS and GWAS data, we randomly selected 345 individuals (approximately two-thirds of 464) as the study sample. Software package IMPUTE2 (version 2.2.2) [4,5] was used to impute SNPs on chromosome 3 that were represented on the reference panel but not in the GWAS data. We compared results from 7 reference panels: (a) all 1094 individuals from 1000 Genomes phase 1 with African, Asian, European, and American ancestries on approximately 37 million SNPs (1000G-all); (b) 120 randomly selected individuals from 1000 Genomes phase 1 (1000G-random); (c) 246 individuals with the African ancestry (1000G-AFR), 286 individuals with the Asian ancestry (1000G-ASN), 381 individuals with the European ancestry (1000G-EUR), and 181 individuals with the American ancestry (1000G-AMR) from 1000 Genomes phase 1; and (d) 119 individuals with GAW18 WGS data not selected for the study sample on approximately 24 million SNPs (GAW18-WGS). It is worth mentioning that 1000G-random was generated through 120 individuals selected randomly from 1000G-all, which was intended to match the size of GAW18-WGS and make the 2 reference panels more comparable. Also, the sampling origins of 1000G-AFR were, with sample counts in parentheses, African Ancestry in Southwest US (61), Luhya in Webuye, Kenya (97), and Yoruba in Ibadan, Nigeria (88); the sampling origins of 1000G-ASN were Han Chinese in Beijing, China (97), Han Chinese South (100), and Japanese in Tokyo, Japan (89); the sampling origins of 1000G-EUR were Utah residents (CEPH) with Northern and Western European ancestry (87), Finnish from Finland (93), British from England and Scotland (89), Iberian populations in Spain (14), and Toscani in Italy (98); and the sampling origins of 1000G-AMR were Colombian in Medellin, Colombia (60), Mexican Ancestry in Los Angeles, CA (66), and Puerto Rican in Puerto Rico (55).

To determine the order of genetic similarity of different reference panels to the Mexican American study sample, we selected a set of SNPs within which no pair were correlated with linkage disequilibrium *r^2 ^*>0.2. For this set of nearly independent SNPs, we then computed genome-wide identity-by-state (IBS) (i.e., the sum of the number of IBS alleles at each locus divided by twice the number of loci) between each pair of individuals in each reference panel along with the study sample. Each reference panel's degree of genetic similarity to the study sample can be represented by its average genome-wide IBS over all pairs of individuals.

We used the imputed reference panel genotypes to evaluate the success of imputation based on GWAS data. The imputation software IMPUTE2 does not estimate the best-guess genotype of a SNP; instead, it estimates the distribution of the genotype, providing probabilities for each probable genotype. Therefore, a certain number of maximum probabilities will exceed a threshold (e.g., 0.9), and among these we ask what percentage of the best-guess imputed genotypes disagree with the observed WGS genotypes. In the meantime, we can calculate the percentage of all imputed genotypes for which no probability exceeds the threshold (i.e., no call is made). Under a given threshold, the above percentage of discordances between imputed genotype calls and observed WGS calls was used as a surrogate for the imputation error rate, and the percentage of genotypes for which no call was made was used as a surrogate for the missing genotype rate. Note that the presented approach assumed no errors in the WGS data.

### Data transformation and program settings

WGS data in GAW18 was provided in vcf files. Software package VCFtools (version 0.1.9) [9] was used to transform vcf files to PLINK [10,11] PED format--files with suffixes ".ped" and ".map." Software package GTOOL (version 0.7.5) [12] was then used to convert PLINK PED files to the file format used by IMPUTE2--files with suffix ".gen." When performing IMPUTE2, we split chromosome 3 into 26 nonoverlapping analysis chunks (with each chunk spanning ~5 megabases [Mb]), and adopted "prephasing" functionality. These are suggested in the webpage of IMPUTE 2 to speed up the analysis. We also performed a postimputation filtering to exclude imputed SNPs that had "info" metric for imputation certainty <0.5.

## Results

We selected 55,389 nearly independent SNPs that exist in all reference panels and the study sample. The average genome-wide IBSs for the study sample for 1000G-AFR, 1000G-all, 1000G-random, 1000G-ASN, 1000G-EUR, 1000G-AMR, and GAW18-WGS are 0.655, 0.677, 0.678, 0.682, 0.683, 0.688, and 0.692, respectively, which represents the degrees of genetic similarity to the study sample from farthest to closest.

**Figure 1:**
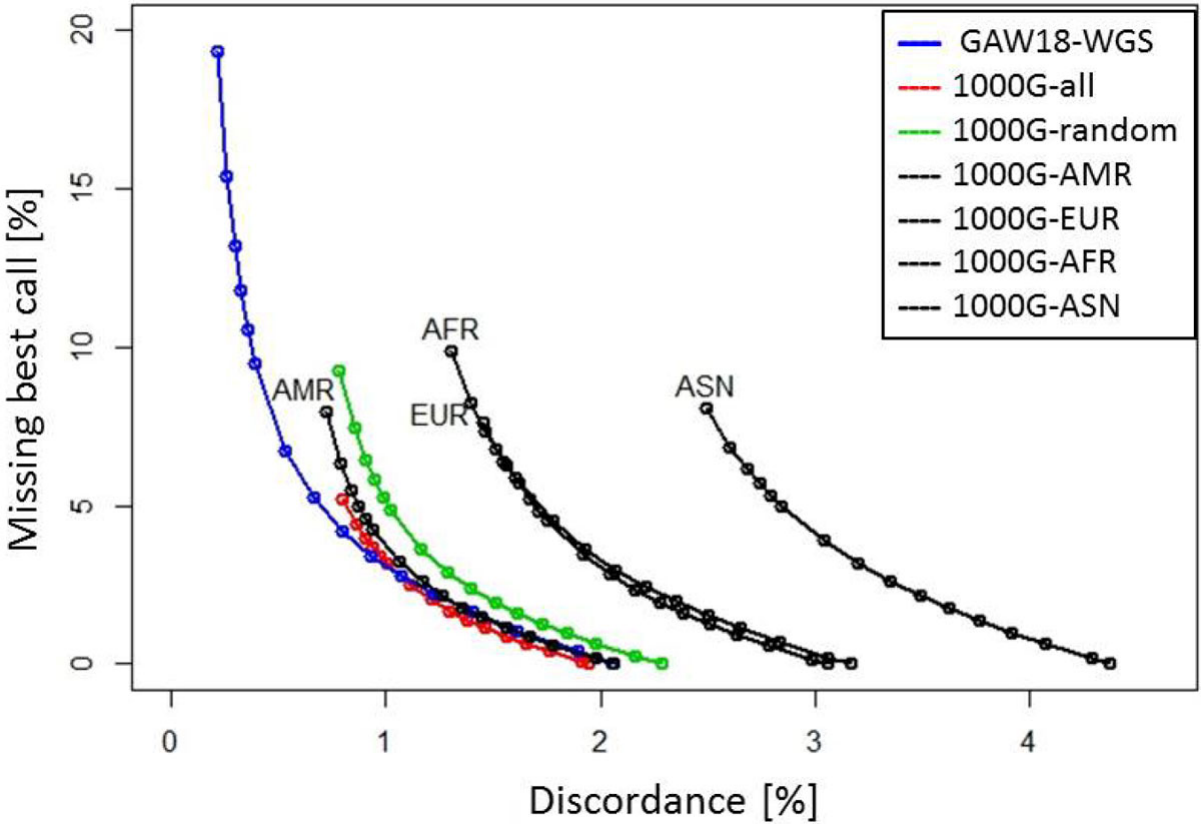
**Discordance percentages versus no-call percentages for different reference panels**. Each line on the plot is generated by repeating calculations of 2 percentages for calling thresholds, ranging from 0.33 to 0.99 for a reference panel.

For all reference panels, discordance and missing rates are calculated based on the 773,165 SNPs (on chromosome 3) that are present in both 1000 Genomes phase 1 and WGS data, but not present in the GWAS data. Figure 1 shows discordance percentages (x-axis) versus no-call percentages (y-axis) for different reference panels. Each line on the plot was generated by repeating calculations of 2 percentages for calling thresholds, ranging from 0.33 to 0.99 for a reference panel.

Clearly, for thresholds >0.45, the GAW18-WGS reference panel creates lower imputation error rates than the reference panels from 1000 Genomes phase 1 do. However, GAW18-WGS can have higher missing genotype rates than 1000 Genomes references for most thresholds. These results may indicate that a reference panel that closely matches the ancestry of the study population can increase imputation accuracy, but this can also risk losing diversity and thus make it harder to identify haplotype sharing with simple models, thereby resulting more missing genotype calls.

The reference panel made up of 181 individuals with the American ancestry from 1000 Genomes phase 1 (1000G-AMR) created imputation error rates and missing genotype rates very close to those based on the reference panel made up of all 1094 individuals from 1000 Genomes phase 1 (1000G-all). However, when adopting reference panels not containing the 181 Americans, the produced imputation error rates and missing genotype rates are much larger than those based on the 1000G-all panel. Despite the much less diversity of the 1000G-AMR panel than that of the 1000G-all panel, these results show that the 181 Americans are what's important in genotype imputation. We can also find that, for the admixed study sample, reference panels with well-defined single ancestry (1000G-AFR, 1000G-ASN, and 1000G-EUR) are not appropriate. The composite reference panels like 1000G-all and 1000G-random should be adopted.

Interestingly, although the 1000G-ASN is, on average, more genetically similar to the study sample than 1000G-AFR, the imputation error rates for 1000G-ASN are much larger than those for 1000G-AFR. Also, 1000G-EUR has higher genetic similarity to the study sample but similar imputation error rates, comparing with 1000G-AFR. This may be a result of the inclusion of people with African ancestry from the southwestern United States in 1000G-AFR.

The reference panel made up of 120 randomly selected individuals from 1000 Genomes phase 1 (1000G-random) has slightly worse imputation error rates and missing genotype rates than those based on the 1000G-all and 1000G-AMR panels. Although large sample size can reduce imputation error and missing genotype, the improvement can be limited.

## Discussion

Reference panels used in genotype imputation can be obtained from publicly available databases or from a 2-stage approach in which a subset of individuals in the study population is selected for whole genome sequencing. Two strategies create reference panels with different degrees of genetic similarity to the study sample. Using WGS and GWAS data of GAW18, this study explores genotype imputation accuracy among reference panels generated by these 2 strategies. Results show that a reference panel that closely matches the ancestry of the study population can increase imputation accuracy, but it can also result more missing genotype calls. Having a reference panel with larger size can reduce imputation error and missing genotype, but the improvement can be limited. We also find that, for the admixed study sample, the simple selection of a single best-reference panel among a HapMap African, European, or Asian population is not appropriate. The composite reference panel combining all available reference data should be used.

Based on our results, the 2-stage approach for genotype imputation is recommended if the extra cost is affordable. When adopting reference panels from publicly available databases, one must include the individuals that most closely match the ancestry of the study population as the reference panel. When the computational burden is a big concern, only the best-matched individuals are included in the reference panel. In the situation with no clear reference matches, larger and more diverse reference collections are recommended as long as appropriate calling thresholds are used.

## Competing interests

The authors declare that they have no competing interests.

## Authors' contributions

GHH designed the overall study, conducted statistical analyses, and drafted the manuscript. YCT conducted statistical analyses and drafted the manuscript. All authors read and approved the final manuscript.
